# Assessment of Treatment Outcome in Chronic Myelogenous Leukemia Patients on Tyrosine Kinase Inhibitors: Insight From a Resource Limited Setting

**DOI:** 10.1002/cam4.70635

**Published:** 2025-02-07

**Authors:** Wude Yewondwosen Awlachew, Abel Tenaw Tasamma, Firehiwot Abebe Mengistie, Zekarias Tadele Alemineh, Samuel Tesfaye Tefera

**Affiliations:** ^1^ Department of Internal Medicine Addis Ababa University Addis Ababa Ethiopia; ^2^ Department of Internal Medicine Axum University Axum Ethiopia; ^3^ Department of Internal Medicine Debrebirhan University Debre Birhan Ethiopia

**Keywords:** chronic myelogenous leukemia, TASH, treatment pattern

## Abstract

**Background:**

Chronic myeloid leukemia (CML) is a hematologic malignancy characterized by the translocation t(9;22)(q34;q11.2), which results in a constitutively active tyrosine kinase. The introduction of tyrosine kinase inhibitors (TKIs) has significantly altered the disease course for patients with BCR‐ABL1+ CML globally.

**Objective:**

This study aimed to evaluate the treatment outcomes of CML patients in Ethiopia.

**Methodology:**

This was a single‐center, cross‐sectional study conducted on 330 patients diagnosed with CML, who were on TKI therapy and receiving follow‐up at Tikur Anbessa Specialized Hospital (TASH). Data were collected from electronic medical records using a structured data abstraction tool. Chi‐square statistics and binary logistic regression were employed to examine the associations between categorical variables, with statistical significance set at a *p* value < 0.05. A 95% confidence interval was used.

**Results:**

The median age of patients was 37.0 years (interquartile range [IQR]: 29.0–49.3), and 185 (56.1%) of the patients were male. At diagnosis, 92.1% were in the chronic phase of CML, while 9 (2.7%) were in blast crisis. The complete hematologic response (CHR) rate at 3 months was 90.9% (291/320), while the CHR rate beyond 3 months was 90.2% (258/286). Multivariable logistic regression analysis showed that the likelihood of not achieving a complete/partial hematologic response was higher among those diagnosed in the advanced phase of CML (AOR: 6.114, 95% CI: 2.210, 16.910) and among patients requiring a treatment change (AOR: 5.765, 95% CI: 2.460, 13.512).

**Conclusion:**

The median age of CML patients in our study was notably young. The 3‐month and overall hematologic responses were excellent. The initial phase of CML at diagnosis and treatment switch was associated with the 3‐month CHR, while only treatment switch was associated with the overall CHR.

## 1 Introduction

Chronic myeloid leukemia (CML) is a clonal myeloproliferative stem cell disorder characterized by the dysregulated production and uncontrolled proliferation of mature and maturing granulocytes, which show relatively normal differentiation. CML is associated with the fusion of the BCR gene on chromosome 22 and the ABL1 gene on chromosome 9, resulting in the BCR::ABL1 fusion gene, which encodes a non‐receptor tyrosine kinase that becomes deregulated and constitutively active. This deregulated tyrosine kinase is implicated in the pathogenesis of CML [1].

According to the Global Burden of Disease Study 2019, CML occurs at an incidence of 1–2 cases per 100,000 people per year and accounts for 15%–20% of adult leukemia cases. The median age at diagnosis in the Western world is 50 years. In Ethiopia, CML is the most common type of leukemia, accounting for more than 50% of leukemia cases, with a median age of presentation of 37 years [2].

CML typically progresses from a relatively indolent (chronic stable) phase, present in approximately 85% of patients at diagnosis and easily controlled with oral agents, to a more aggressive (accelerated) phase, during which disease control becomes more challenging. This phase ultimately leads to the blast phase [3]. The treatment of CML has evolved significantly with the introduction of tyrosine kinase inhibitors (TKIs). These agents have enabled long‐term control of CML in the majority of patients, making them the first‐line treatment for all diagnosed cases of CML [4].

The goal of therapy for all CML patients treated with TKIs is to achieve clinical, cytogenetic, and molecular remission, maintain long‐term disease control, and prevent disease progression while optimizing quality of life by minimizing treatment‐related toxicity. In clinical trials, progression rates during TKI treatment have been reduced to less than 4% per year [5, 6, 7].

The treatment outcomes of CML have been revolutionized with the advent of TKIs. In developed countries, these outcomes have been well‐studied and documented, with real‐world treatment outcome studies and registries showing that results are consistent with clinical trials. However, data from developing countries remains limited. In Ethiopia, there have been no previous studies evaluating the outcomes of CML patients on TKI therapy. This study aims to evaluate the treatment outcomes and factors influencing these outcomes in patients with CML on TKI therapy treated at Tikur Anbessa Specialized Hospital (TASH) from October 2018 to 2022.

By assessing treatment outcomes and identifying predictive factors, this study aims to fill a crucial knowledge gap in Ethiopia, where no local research exists on this topic. Understanding the gaps in management and the factors affecting outcomes will help advocate for improvements at both institutional and governmental levels, ultimately improving patient care. This research will also serve as a useful reference for future studies, health planning, treatment funding, and research on similar disease entities.

## 2 Methods and Materials

### 2.1 Study Setting

This study was conducted at the outpatient hematology clinic of TASH. Patients from all regions of Ethiopia are referred to this hospital to be enrolled in the Glivec International Patient Assistance Program (GIPAP), which provides free access to treatment with imatinib and second‐generation TKIs. These patients are followed closely in the outpatient clinics of the Department of Internal Medicine's Hematology Division.

### 2.2 Study Design and Period

A hospital‐based, cross‐sectional study was conducted to assess the treatment outcomes of CML patients on TKI therapy in Ethiopia. The study was conducted from October 2022 to November 2022.

### 2.3 Study Population

All CML patients who began follow‐up at TASH between October 2018 and 2022 and had been on TKI therapy for at least 3 months during the study period.

### 2.4 Eligibility Criteria

#### 2.4.1 Inclusion Criteria

- Diagnosis of CML
- Patients who started TKI therapy
- Patients on TKI therapy for more than 3 months

#### 2.4.2 Exclusion Criteria

- Incomplete data
- CML patients not started on TKI

### 2.5 Sample Size Determination and Sampling Method

The sample size will be calculated using the single proportion formula

$$\frac{n=Z_{\alpha /2}^{2}\mathrm{pq}}{d^{2}}$$

where *Z α*/2 is standard normal variant (at 5% type 1 error (*p* < 0.05)) it is 1.96. *d* = margin of error was taken as 0.05. *p* = 0.5 (no local data is available). *q* = 1−*p*: the probability of non‐occurrence of the event of interest.

The calculated sample size is 384 patients.

Since the population size is less than 10,000, we need additional correction with

$$n=\frac{n0}{1+\frac{n0}{N}}$$

where *n*, final sample size. *n*_0_, initial sample size (384). *N*, source population size (1800).

The calculated sample size is 316 patients and 10% loss was added with resulting total size of 347 patients.

### 2.6 Sampling Technique

Systematic random sampling was used to select participants.

### 2.7 Study Variables

#### 2.7.1 Dependent Variables

- Treatment outcome at the 3rd month
- Overall treatment outcome

#### 2.7.2 Independent Variables

- Sociodemographic factors
- Sokal score
- Phase of CML
- Comorbidities
- Interval from diagnosis to starting TKI therapy
- Therapy duration
- CBC profile
- Change in TKI

### 2.8 Data Collection Procedure and Data Quality

A structured data abstraction tool, adapted from other relevant studies, was created using Google Forms. The HMIS registry was used to retrieve patient medical record numbers (MRNs), and each patient's electronic medical record (EMR) was reviewed. Data were collected by the investigator, interns, and staff nurses. Intensive training for data collectors was conducted 1 week prior to the survey, and the lead investigator continuously monitored the process, offering guidance. Pretesting of the questionnaire was performed on 5% of the sample. The data were verified for accuracy before entering.

### 2.9 Data Analysis

Data were checked for completeness, edited, coded, and exported to SPSS version 20.6 for cleaning and analysis. Frequencies and proportions were used to describe study participants and sociodemographic characteristics. Continuous variables were expressed as means ± SD, and categorical variables were presented as percentages. Continuous variables were compared using the Student's *t*‐test, while categorical variables were compared using the chi‐square test. A *p* value of < 0.05 was considered statistically significant for all tests.

## 3 Result

### 3.1 Sociodemographic Characteristic

A total of 330 participants were included in the study, resulting in a 95.1% response rate. Seventeen patients were excluded: four were pregnant, and the remaining 13 had incomplete medical records. The median age of the patients was 37.0 years, with an interquartile range (IQR) of 29.0–49.0. Of the participants, 185 (56.1%) were male. Approximately one‐third (105, 31.8%) were from the Oromia region, and 73 (22.1%) were from Addis Ababa. Two‐thirds (221, 67%) of the patients resided within a 400 km radius of the hospital (See Table 1).

**TABLE 1:** Sociodemographic characteristic of chronic myelogenous leukemia patients on tyrosine kinase inhibitors on follow‐up At Tikur Anbessa Specialized Hospital, Addis Ababa; Ethiopia, 2022.

Variable	Response	Frequency	Percentage
Age	Median (IQR)	37.0 (29.0–49.3)	
	≤ 30 years	96	29.1
	31–50 years	156	47.3
	≥ 51 years	78	23.6
Sex	Male	185	56.1
	Female	145	43.9
Region	Addis Ababa	73	22.1
	Oromia	105	31.8
	Amhara	81	24.5
	SNNPR	44	13.3
	Others^a^	27	8.2
Average distance in km from TASH	< 100 km	87	26.4
	101–200 km	35	10.6
	201–300 km	55	16.7
	301–400 km	44	13.3
	401–500 km	60	18.2
	> 500 km	49	14.8

^a^ Nine—Somalia, six—Afar, five—Benishangul, two—Harari, two—Dire Dawa, two—Tigray, and one—Gambella.

### 3.2 Clinical Characteristics of Patients at Diagnosis

Forty‐three patients (13%) had one or more comorbid conditions. The three most common comorbidities were hypertension (12, 3.6%), cardiac illnesses (8, 2.4%), and human immunodeficiency virus (HIV) infections (7, 2.1%). Additionally, 46 patients (13.9%) were taking medications other than TKI. The two most frequently prescribed medications were antidiabetic drugs (9, 2.7%) and highly active antiretroviral therapy (HAART) (7, 2.1%) (See Table 2).

**TABLE 2:** Comorbidities and medication intake of chronic myelogenous leukemia patients on tyrosine kinase inhibitors on follow‐up at Tikur Anbessa Specialized Hospital, Addis Ababa; Ethiopia, 2022.

Variable	Response	Frequency	Percentage
Comorbidity	Yes	43	13.0
	No	287	87.0
Chronic hepatitis B	Yes	4	1.2
	No	326	98.8
Cardiac illnesses	Yes	8	2.4
	No	322	97.6
Hypertension	Yes	12	3.6
	No	318	96.4
Diabetes mellitus	Yes	7	2.1
	No	323	97.9
HIV/AIDS	Yes	7	2.1
	No	323	97.9
Other comorbidities^a^	Yes	12	3.6
	No	318	96.4
Medication intake other than TKI	RAAS inhibitors	4	1.2
	Calcium channel blockers	6	1.8
	HAART	7	2.1
	Diuretics	5	1.5
	Antidiabetic	9	2.7
	Others	15	4.6

^a^ One—chronic hepatitis C, one—gouty arthritis, one—breast cancer, one—ischemic stroke, one—pleural effusion, one—tb lymphadenitis, one—toxic goiter, one—hypothyroidism, and two cases of nephrolithiasis and seizure disorder each.

**FIGURE 1:**
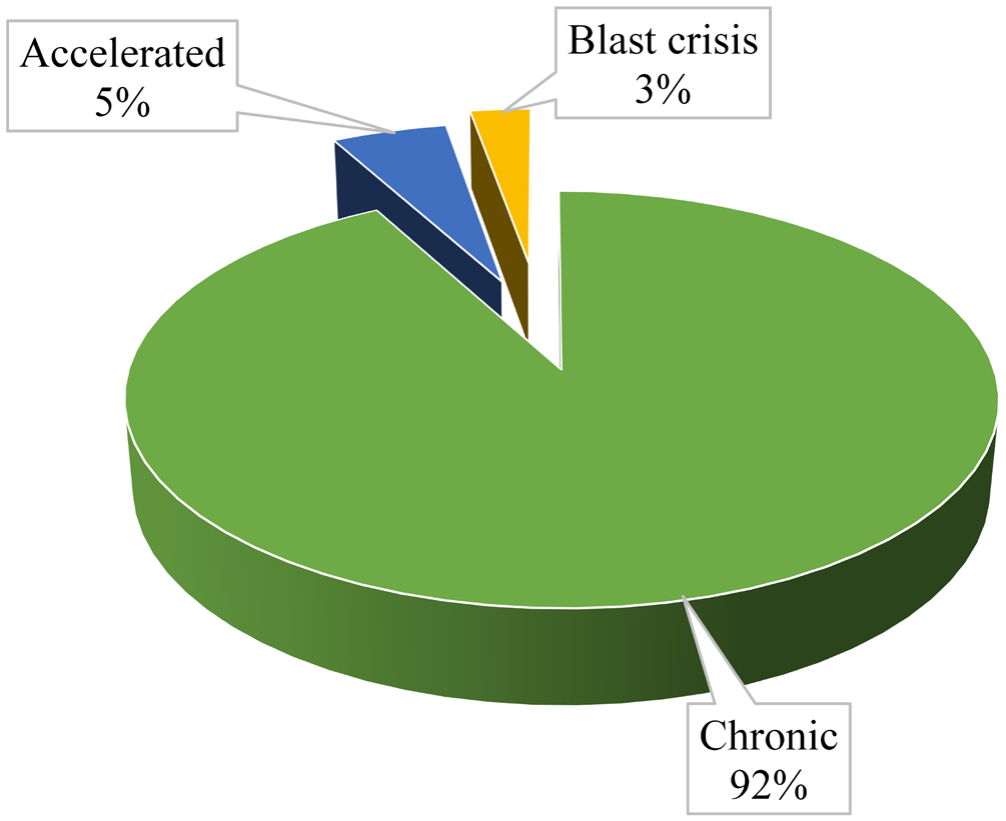
Phase of chronic myelogenous leukemia during initial diagnosis among patients on tyrosine kinase inhibitors on follow‐up at Tikur Anbessa Specialized Hospital, Addis Ababa; Ethiopia, 2022.

Most (304, 92.1%) of the patients in the study were in chronic phase of CML during diagnosis and only 9 (2.7%) were in blast crisis (See Figure 1).

Ninety percent (297) of the patients had a palpable spleen (splenomegaly) at the time of their initial diagnosis of CML, with 200 patients (60.6%) exhibiting significant splenomegaly (≥ 8 cm below the right costal margin). The median (IQR) spleen size below the right costal margin was 10 (6–15) centimeters.

Regarding baseline laboratory studies, the median (IQR) total white blood cell (WBC), basophil, and platelet counts were 196.0 (116.5–291.8) × 10^3^ cells/mm^3^, 2.3 (1.0–4.7) × 10^3^ cells/mm^3^, and 302.5 (211.0–450.0) × 10^3^ cells/mm^3^, respectively. The median (IQR) hemoglobin concentration was 10.0 (8.4–11.4) g/dL.

Using the Sokal score, 147 patients (44.5%) were classified as high risk, 146 (44.2%) as intermediate risk, and the remaining 37 patients (11.2%) were low risk.

### 3.3 Treatment Profile and Outcome of Patients

The median (IQR) duration from diagnosis to initiation of TKI treatment was 14 (0–28.3) days, and the median duration of TKI use was 16.0 (9.0–24.0) months. The initial TKI administered was daily imatinib in nearly all patients (319, 96.7%). However, all patients in blast crisis (9, 2.7%) and three patients in the accelerated phase were started on nilotinib. A total of 292 patients (88.5%) were treated with only one TKI, while 35 patients (10.6%) used two types of TKIs, and three patients (0.9%) used three types of TKIs (See Table 3).

**TABLE 3:** Summary of treatment of chronic myelogenous leukemia patients on tyrosine kinase inhibitors on follow‐up at Tikur Anbessa Specialized Hospital, Addis Ababa; Ethiopia, 2022.

Variable	Response	Frequency	Percentage
Initiation TKI medication	Imatinib	319	96.7
	Nilotinib	11	3.3
Was there dose escalation	Yes	9	2.7
	No	321	97.3
Was there change of TKI	Yes	38	11.5
	No	290	87.9
Second‐line TKI medications used	Nilotinib	23	7.0
	Bosutinib	7	2.1
	Dasatinib	11	3.3
	Ponatinib	4	1.2
What was the reason for change for TKI	Intolerance	8	2.4
	Treatment failure/disease progression	45	13.6
Number of TKI's used	One	292	88.5
	Two	35	10.6
	Three	3	0.9
Duration of TKI treatment	Median (IQ)	16.0 (9.0–24.0)	
	≤ 9 months	83	25.2
	9–18 months	98	29.7
	> 18 months	149	45.2

At the time of data collection, the majority (281, 85.2%) of patients remained on the same dose of imatinib. Nine patients (2.7%) required an imatinib dose escalation, and 40 patients (12.1%) were switched to a second‐line TKI. Nilotinib and dasatinib were the two most commonly used second‐line TKIs, accounting for 23 patients (57.5%) and 11 patients (27.5%), respectively. Treatment failure or disease progression was the primary reason for switching to second‐line TKIs. BCR‐ABL kinase domain mutation analysis was conducted for 15 patients (33.3%) with treatment failure or disease progression. No mutation was detected in eight patients, three patients had mutations (two T315I and one C.749G>A [p.G250E], C.757T>C [p.Y253H]), two patients exhibited imatinib resistance, and one patient each showed intermediate sensitivity to nilotinib and dasatinib.

Among the 320 patients who visited the hematology clinic at the 3‐month mark after initiating TKI treatment, 291 patients (90.9%) achieved a complete hematologic response (CHR), 26 patients (8.1%) achieved a partial hematologic response, and three patients (0.9%) did not achieve any hematologic response. Early molecular response (EMR), measured as the BCR::ABL1 percentage, was assessed for only six patients (1.9%) at the 3‐month mark. Of these, five patients achieved an optimal response (≤ 10%), while one patient experienced failure (45%).

After a median follow‐up period of 16 months, 286 patients (86.7%) remained in follow‐up. Of these, 90.2% (258/286) were in complete hematologic response (CHR), with 242 patients having an unknown major molecular response (MMR) status and 16 patients maintaining MMR. Twenty‐three patients (8.0%) failed to achieve any response, and five patients (1.7%) died. The remaining 44 patients (13.3%) were lost to follow‐up with an unknown response status. The majority of these patients (34, 77.3%) lived more than 200 km away from the hospital.

### 3.4 Predicting Factors Associated With Treatment Outcome

#### 3.4.1 Predicting Factors Associated With Hematologic Response at 3 Months

Eleven independent variables were examined for their potential association with CHR at the end of 3 months through univariate logistic regression analysis. The variables found to be significantly associated with 3‐month CHR (*p* value < 0.05) and included in the multivariable logistic regression were the phase of CML at diagnosis and the need for a treatment change. In the multivariable logistic regression analysis, both the phase of CML at diagnosis and the requirement for a treatment change were significantly associated with CHR at the end of 3 months of TKI treatment.

Multivariable logistic regression analysis showed that the likelihood of being hematologically nonresponsive or partially responsive was 6 times higher among patients diagnosed with accelerated phase CML compared to those in chronic phase (AOR: 6.114, 95% CI [2.210, 16.910]). Patients who required a treatment switch had 5.8 times higher odds of not achieving CHR at the third month compared to those who did not require a treatment switch (AOR: 5.765, 95% CI [2.460, 13.512]) (See Table 4).

**TABLE 4:** Binary logistic regression on determining factors associated with 3‐month CHR of chronic myelogenous leukemia patients with tyrosine kinase inhibitors on follow‐up at Tikur Anbessa Specialized Hospital, Addis Ababa; Ethiopia, 2022.

Variable	3‐month complete response		COR (95% CI)	*p*	AOR (95% CI)	*p*
	Yes (%)	No (%)				
Age in years						
≤ 30 years	87 (29.9)	7 (24.1)	1			
31–50 years	134 (46.0)	16 (55.2)	1.484 (0.587, 3.755)	0.405		
≥ 51 years	70 (24.1)	6 (20.7)	1.065 (0.342, 3.314)	0.913		
Sex						
Male	161 (55.3)	18 (62.1)	1.321 (0.603, 2.896)	0.487		
Female	130 (44.7)	11 (37.9)	1			
Comorbidity						
Yes	38 (13.1)	3 (10.3)	0.768 (0.222, 2.662)	0.678		
No	253 (86.9)	26 (89.7)	1			
Phase of CML at diagnosis						
Accelerated	15 (5.2)	9 (31.0)	8.280 (3.225, 21.257)	< 0.001	6.114 (2.210, 16.910)	< 0.001
Chronic	276 (94.8)	20 (69.0)	1		1	
Sokal score						
Low risk (< 0.8)	32 (11.0)	4 (13.8)	1			
Intermediate risk (0.8–1.2)	134 (46.0)	9 (31.0)	0.537 (0.156, 1.855)	0.326		
High risk (> 1.2)	125 (43.0)	16 (55.2)	1.024 (0.320, 3.274)	0.968		
WBC count at diagnosis						
≤ 100	58 (19.9)	9 (31.0)	1			
101–300	170 (58.4)	12 (41.4)	0.455 (0.182, 1.135)	0.455		
Above 300	63 (21.6)	8 (27.6)	0.818 (0.296, 2.263)	0.818		
Platelet count at diagnosis						
≤ 145	29 (10.0)	6 (20.7)	1			
146–450	190 (65.3)	17 (58.6)	0.432 (0.158, 1.187)	0.104		
Above 450	72 (24.7)	6 (20.7)	0.403 (0.120, 1.352)	0.141		
Hemoglobin at diagnosis						
≤ 10 g/dL	209 (71.8)	19 (65.5)	1			
> 10 g/dL	82 (28.2)	10 (34.5)	1.341 (0.598, 3.007)	0.476		
Basophil						
≤ 1%	75 (25.8)	6 (20.7)	1			
> 1%	216 (74.2)	23 (79.3)	1.331 (0.522, 3.394)	0.549		
Delay from diagnosis to treatment						
≤ 2 weeks	165 (56.7)	14 (48.3)	1			
> 2 weeks	126 (43.3)	15 (51.7)	1.403 (0.653, 3.013)	0.385		
Treatment switch						
Yes	34 (11.7)	14 (48.3)	7.055 (3.134, 15.881)	< 0.001	5.765 (2.460, 13.512)	< 0.001
No	257 (88.3)	15 (51.7)	1		1	

Abbreviations: AOR, adjusted odds ratio; COR, crude odds ratio.

#### 3.4.2 Predicting Factors Associated With Overall Complete Hematologic Remission

Of all the variables included in the univariate and multivariable binary logistic regression analysis (*p* < 0.05), only treatment switch was significantly associated with complete hematologic remission. The study found that a good response to the first initiated TKI and not requiring a treatment switch were predictive of CHR success (AOR = 28.214, 95% CI: [11.093, 71.759]).

In other words, patients who switched to second‐line TKIs had a 28 times lower chance of achieving CHR compared to those who did not switch (See Table 5).

**TABLE 5:** Binary logistic regression on determining factors associated with overall CHR of chronic myelogenous leukemia patients with tyrosine kinase inhibitors on follow‐up at Tikur Anbessa Specialized Hospital, Addis Ababa; Ethiopia, 2022.

Variable	Complete CHR response		COR (95% CI)	*p*	AOR (95% CI)	*p*
	Yes (%)	No (%)				
Age in years						
≤ 30 years	76 (29.5)	5 (17.9)	0.997 (0.257, 3.873)	0.996		
31–50 years	121 (46.9)	19 (67.9)	0.418 (0.136, 1.282)	0.127		
≥ 51 years	61 (23.6)	4 (14.3)	1			
Sex						
Male	142 (55.0)	15 (53.6)	1.061 (0.485, 2.319)	0.882		
Female	116 (45.0)	13 (46.4)	1			
Comorbidity						
Yes	33 (12.8)	6 (21.4)	1			
No	225 (87.2)	22 (78.6)	1.860 (0.702, 4.924)	0.212		
Phase of CML at diagnosis						
Accelerated	16 (6.2)	3 (10.7)	1			
Chronic	242 (93.8)	25 (89.3)	1.815 (0.495, 6.660)			
Sokal score						
Low risk (< 0.8)	35 (13.6)	2 (7.1)	2.593 (0.568, 11.837)	0.219		
Intermediate risk (0.8–1.2)	115 (44.6)	10 (35.7)	1.704 (0.741, 3.917)	0.210		
High risk (> 1.2)	108 (41.9)	16 (57.1)	1			
WBC count at diagnosis						
≤ 100	61 (23.6)	5 (17.9)	2.241 (0.705, 7.120)	0.171		
101–300	148 (57.4)	14 (50.0)	1.942 (0.791, 4.764)	0.147		
Above 300	49 (19.0)	9 (32.1)	1			
Platelet count at diagnosis						
≤ 145	29 (11.2)	1 (3.6)	3.803 (0.454, 31.855)	0.218		
146–450	168 (65.1)	19 (67.9)	1.160 (0.483, 2.786)	0.741		
Above 450	61 (23.6)	8 (28.6)	1			
Hemoglobin at diagnosis						
≤ 11 g/dL	179 (69.4)	21 (75.0)	1			
> 11 g/dL	79 (30.6)	7 (25.0)	1.324 (0.541, 3.242)	0.539		
Basophil % at diagnosis						
≤ 1%	66 (25.6)	9 (32.1)	0.726 (0.313, 1.683)	0.455		
> 1%	192 (74.4)	19 (67.9)	1			
Delay from diagnosis to treatment						
≤ 2 weeks	146 (56.6)	19 (67.9)	0.617 (0.269, 1.417)	0.255		
> 2 weeks	112 (43.4)	9 (32.1)	1			
Treatment switch						
Yes	21 (8.1)	20 (71.4)	1		1	
No	237 (91.9)	8 (28.6)	28.214 (11.093, 71.759)	<0.001	28.214 (11.093, 71.759)	<0.001
Duration on TKI treatment						
≤ 9 months	54 (20.9)	3 (10.7)	2.252 (0.630, 8.040)	0.212		
9–18 months	76 (29.5)	9 (32.1)	1.056 (0.445, 2.506)	0.902		
> 18 months	128 (49.6)	16 (57.1)	1			
Hematologic response at 3 months on TKI						
Yes	19 (7.5)	3 (10.7)	1.478 (0.409, 5.345)	0.551		
No	234 (92.5)	25 (89.3)	1			

## 4 Discussion

In this cross‐sectional study, we evaluated the treatment outcomes of CML patients on TKIs and the determinant factors for these outcomes at Tikur Anbessa Specialized Hospital, Addis Ababa, Ethiopia.

Regarding the clinical and biological features, the median age of our study participants was 37.0 years, with most patients falling in the 31–50 years age group. This is consistent with a study conducted in Ethiopia [2] and many other African studies, where the median age ranged from 34 to 40 years [8, 9]. This younger age group is notably different from the median age of 50 years observed in major imatinib clinical trials [10, 11]. This younger age distribution may reflect healthcare system biases favoring younger patients with symptomatic disease, or it may indicate biological differences that warrant further investigation.

Other studies have shown that sex can be a determinant of treatment outcomes in CML patients on TKI therapy [12, 13]. However, our study found no association between overall patient outcomes and sex. This could be due to the smaller sample size in our study.

Due to the long time to the first medical visit, most of the patients were symptomatic, with 90% presenting with splenomegaly at the time of initial diagnosis. This finding is comparable to a study conducted in Togo (80%) [14], whereas in high‐income countries, 50% of CML patients are asymptomatic at diagnosis [15].

Since access to TKIs is severely restricted in our country, all treatment naïve chronic phase CML patients were started on imatinib regardless of their risk profile. The switch to nilotinib or dasatinib occurred regardless of mutational analysis due to the unaffordability of the test, for suboptimal responses, or for patients who failed to achieve a response with imatinib. The lack of testing for mutations associated with nilotinib resistance could bias assessment of treatment response for the drug as patients with resistance maybe wrongfully initiated on the medication. However, the price of the test is quite expensive and not affordable to the overwhelming majority of our patients. This is the reason mutational analysis isn't routinely done at the initiation of TKIs in our cohort. Additionally, all patients in blast crisis and some in accelerated phase of CML were initiated directly on nilotinib. These patients exhibited a poor 3‐month CHR, as observed in other studies [12]. This could be attributed to the aggressiveness of the disease phase and the late diagnosis of these patients. Although our study lacked comprehensive molecular assessments, other studies have shown that the level of BCR‐ABL transcription was significantly lower at 3, 6, and 12 months in patients switched to second‐line therapies [16, 17].

After 3 months of TKI initiation, 90.9% of our patients achieved CHR, a considerably higher rate compared to the 82.3% reported in a study from Côte d'Ivoire [18]. This result is similar to studies in Tanzania and China, where the 3‐month CHR was 91.3% and 92.1%, respectively [19, 20]. The remaining 10% who did not achieve CHR may have been affected by lower treatment adherence, as reported in a study by Mulu et al. in our population [21]. The unaffordable price of molecular tests means additional cytogenetic aberrations are almost never assessed in our patients at diagnosis or after failed treatment. Although BCR‐ABL MRD testing is performed for some patients, its inconsistent availability limits comprehensive analysis. These factors are also why our study focuses solely on hematologic response.

With an overall median follow‐up of 16 months, 84.6% of patients maintained CHR, a higher rate than that found in similar studies [6, 9]. This suggests that the long‐term outcomes of imatinib therapy remain positive. However, the high CHR rate in our study could also be influenced by the high rate of lost‐to‐follow‐up patients, for whom we were unable to determine the outcomes.

Despite imatinib being the treatment of choice for newly diagnosed chronic phase CML patients, about one‐third of patients will experience poor responses, either due to primary failure or progression after an initial response [21]. Several mechanisms, including point mutations in the BCR‐ABL kinase domain, can cause treatment resistance, leading to poor outcomes [22]. In our study, only 15 patients underwent mutational analysis. We found that treatment failure or disease progression was associated with a change in therapy, which aligns with a study conducted in Iraq [23].

The Sokal score is known to predict the achievement of MMR in CML patients [24]. However, in our study, there was no correlation between achieving CHR and the Sokal score. This may be due to our small sample size, potential information bias related to the retrospective nature of our study, and inconsistent reporting of splenomegaly. Interestingly, a study from Iraq also found no association between the Sokal score and CHR [23].

Initial WBC, hemoglobin, platelet counts, and duration of symptoms were not significant predictors of CHR, suggesting that advanced presentations alone may not account for increased mortality. Biologic differences should be further investigated, as suggested by a study conducted in Rwanda [18]. The use of ΕԼTS score was recommended by a reviewer. We understand that the ΕԼTS (EUΤΟS long‐term survival score) offers the best discrimination for predicting the probability of CML‐specific death. However, we did not analyze factors associated with CML‐specific death because the mortality rate in our cohort was very low (five out of 330, or 1.7%). Additionally, the Sokal score is by far the most commonly used risk assessment tool in our clinical practice. For these two reasons, we chose to use the Sokal score rather than the ΕԼTS to examine factors associated with treatment response.

Most patients who were lost to follow‐up were living more than 200 km away from the hospital. This finding is consistent with Mulu et al., who noted that living in rural areas was a significant factor for nonadherence to treatment [21]. This emphasizes that distance from the treatment center has a significant impact on CML management. Furthermore, the accessibility and affordability of molecular response assessments are critical challenges in managing CML in Ethiopia and other developing countries [12].

Limitations of this study include the retrospective design, which resulted in incomplete data and limited our assessment of initial prognosis, clinical failure, and treatment adherence. Specifically, rates of imatinib discontinuation due to clinical failure were poorly documented. Additionally, the use of different laboratories for the initial assessment of patients' Sokal scores may have led to information bias. Variations in the documentation of physical findings, such as splenomegaly, due to different physicians contributing to the medical records also contributed to inconsistencies.

## 5 Conclusion

In conclusion, the 3‐month and current hematologic responses in CML patients on TKIs at our hospital were found to be very good. The median age of CML patients in our study was strikingly young. The initial phase of CML at diagnosis and treatment switch were the two factors associated with the 3‐month CHR, and only treatment switch was found to be a determinant of current CHR.

These results highlight significant challenges in managing CML in developing countries, including delayed medical visits and high costs of diagnostic tests. These factors may contribute to low response rates to imatinib and increased mortality. Early diagnosis, better identification of weak responders, and the availability of second‐ and third‐line TKIs could significantly reduce mortality in CML patients in our country.

Achieved hematologic responses should be supported and confirmed by molecular responses to better understand treatment outcomes. Therefore, it is crucial to advocate for the importance of recommended tests and collaborate with governmental and nongovernmental organizations to make these tests more affordable and accessible. Additionally, creating treatment centers in various parts of the country will improve accessibility and reduce the number of patients who discontinue follow‐up. Improving healthcare‐seeking behavior and early detection will also contribute to better outcomes for CML patients.

## Author Contributions

**Wude Yewondwosen Awlachew:** conceptualization, writing – original draft, formal analysis, data curation. **Abel Tenaw Tasamma:** writing – review and editing, supervision. **Firehiwot Abebe Mengistie:** methodology, formal analysis, investigation. **Zekarias Tadele Alemineh:** resources, validation, conceptualization. **Samuel Tesfaye Tefera:** investigation, software.

## Ethics Statement

Ethical approval was obtained from the Institutional Review Board (IRB) of Addis Ababa University (AAU), Department of Internal Medicine. Written or verbal consent was not required as the study only used secondary data.

## Conflicts of Interest

The authors declare no conflicts of interest.

## Data Availability

The data that support the findings of this study are available from the corresponding author upon reasonable request.
